# Military Favor toward the Medical Profession

**Published:** 1918-12

**Authors:** 


### Military Favor Toward the Medical Profession.

We have, in common with most medical journals, and in a proper spirit, called attention to the sacrifice demanded of the medical profession, in particular. It is worth while to present the other side of the case.

Excepting the clergy, the medical profession is the only vocation whose services are asked solely as commissioned officers. Even medical students who were bona fide matriculants at the beginning of the state of war, are practically exempt from the draft and first year students who are drafted, will be allowed to serve in the hospital corps and will probably receive non-commissioned rank if at all eligible. A great many men are not afraid of death and without being swash-bucklers, desire military service, some as a matter of pure patriotism, some because they would rather be ancestors than descendants, veterans instead of sons or great great grandsons, some from love of excitement, at least for the sake of getting out of a rut, some to get away from non-peaceful peace conditions of one kind or another. To such men, the extended age limit and liberal allowance for physical defects is a favor. And there have been enough such to render the service of the profession as a whole, entirely voluntary.

Again excepting the clergy, whose status and numbers are quite different, the medical profession is the only vocation which enters military service on the basis of its normal daily work and without the requirement of preliminary military training. Of course, whenever practicable, this preliminary training is given but it is not absolutely required. When required, the medical volunteer undergoes training on full pay instead of at his own expense or on private's pay. In many instances, preliminary training takes the form of post-graduate courses which, before the war, were expensive to the student and sometimes almost unobtainable without special influence. Excepting inevitable records, formalities and peculiar cases, the military service of the physician is, in iself, a preparation for further private practice. The recognition of specialties and subdivision between surgical and medical work renders this particularly true of the present war.

The medical profession called to military service is given higher rank and pay than any other profession or vocation. Yes, we know that every man in a rainbow camp may hope to become a general, just as every native born citizen may hope to become president; and that the physician can not rise beyond major—except by recent changes which will affect only a few, or unless, under extremely favorable circumstances, he is switched off to a career in the Regular Army Medical Corps. But the statement is correct in the average sense; and it refers to ordinary competence in one's own line of work and not to developing unsuspected talents along an entirely different line.

The average physician, called to military service, will have a larger income than in private life. Of course, you and I are making a tremendous financial sacrifice but we speak for the average professional income of the other fellow. If this statement still seems extreme, bear in mind the difference between what we charge on the books and what we collect, between the latter and what is left over after paying professional expenses. There are, of course, men whose military pay will be only a small part of what they have been accustomed to earn but such men ought to be able to live on their accumulations; and their devotion to the country's needs for a year or two or three, is a professional investment.

Statistics show that the mortality and disability of officers is higher than for privates and, contrary to expectation, that the medical officers have the highest risk of all. But the actual increased risk is not very great, indeed the entire military risk is only about half of the expected mortality in peace, and not even so great for the average age of medical officers, since the direct military risk is practically the same for all ages. It must also be considered that the hardships of medical officers can scarcely be so great as those of many of the line officers and that the former are nearest the source of both prophylactic and therapeutic care.

